# Silicone Breast Implants: A Rare Cause of Pleural Effusion

**DOI:** 10.1155/2015/652918

**Published:** 2015-11-26

**Authors:** Imam H. Shaik, Bindu Gandrapu, Fernando Gonzalez-Ibarra, David Flores, Jyoti Matta, Amer K. Syed

**Affiliations:** ^1^Department of Internal Medicine, Jersey City Medical Center, Jersey City, NJ 07302, USA; ^2^Zaporozhye State Medical University, Zaporozhye 69035, Ukraine; ^3^Department of Pulmonary and Critical Care Medicine, Jersey City Medical Center, Jersey City, NJ 07302, USA; ^4^Laureate National Institute of Medicine, Program Director Internal Medicine, Jersey City Medical Center, Jersey City, NJ, USA

## Abstract

Pleural effusions are one of the rarest complications reported in patients with silicone gel filled breast implants. The silicone implants have potential to provoke chronic inflammation of pleura and subsequent pulmonary complications such as pleural effusion. Herein, we report a 44-year-old female who presented with left sided pleural effusion, six weeks after a silicone breast implantation surgery. The most common infectious, inflammatory, and malignant causes of pleural effusion were excluded with pleural fluid cytology and cultures. With recurrent effusion in the setting of recent surgery, the chemical reaction to silicone breast implants was sought and exploration was performed which revealed foreign body reaction (FBR) to silicone material. The symptoms dramatically improved after the explantation.

## 1. Introduction

Breast implant is a kind of medical device used to augment or reconstruct breast size after breast surgery or to correct the shape of the breast for cosmetic purposes. Food and Drug Administration (FDA) has reported 5 to 10 million women with breast implants worldwide, where silicone gel filled implants are used abundantly [1]. American Society of Plastic Surgeons National Clearinghouse of Plastic Surgery Procedural Statistics reports that 296,203 and 93,083 breast augmentation procedures and breast reconstruction procedures, respectively, were carried out in United States in the year 2010 [2]. About half of these implants were silicone breast implants. FDA has acknowledged that benefits of labeled silicone gel filled breast implants weigh over complications and carry reasonable safety and outcome [1].

Most common complications of silicone gel filled breast implants include capsular contracture, reoperation, rupture, wrinkling, asymmetry, scarring, pain, and infection. Pleural effusion is very rare in the setting of intact capsule. The first ever case of pleural effusion caused by the rupture of silicone bag mammary prosthesis was reported by Stevens et al. in 1987 [3]. The present case study reports a middle aged woman with left sided pleural effusion six weeks after the placement of silicone gel filled breast implant. This case is a useful addition to the literature of a rare complication of silicone gel filled breast implant, which should be in a differential diagnosis in patients presenting with unexplained pleural effusions after the breast implants.

## 2. Case Presentation

A 44-year-old female presented to the emergency department with three-week history of left sided pleuritic chest pain associated with worsening shortness of breath. There was no history of associated cough, sputum production, fever, or chills. Her medical history was significant for Phyllodes tumor of the left breast, for which she underwent bilateral mastectomy and later underwent breast augmentation surgery with silicone implants six weeks prior to the presentation. The patient underwent outpatient workup with a CT angiogram of the chest which showed mild inflammatory changes. She was prescribed empiric antibiotics for a possible pneumonia, but her progressions of symptoms despite antibiotics made her visit the ER.

**Figure 1:**
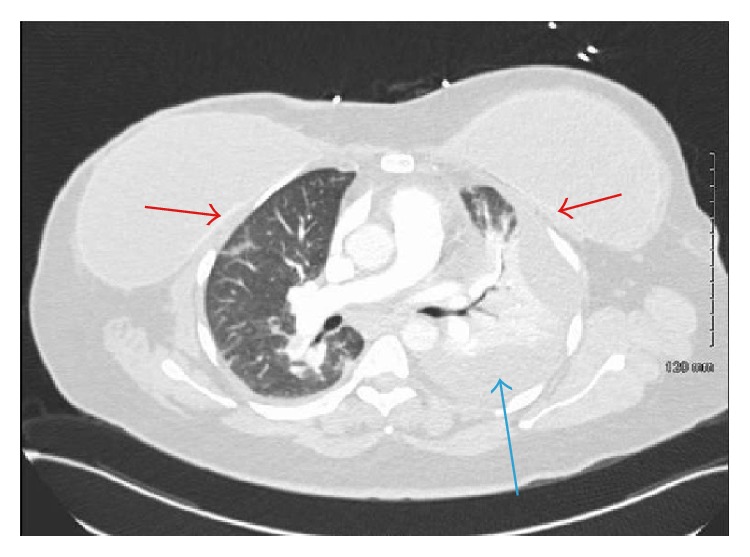
Contrast CT of the thorax showing moderate size left sided pleural effusion (blue arrow) and bilateral silicone implants with intact capsule (red arrows).

On physical examination, she was well built, afebrile, and hemodynamically stable with a heart rate of 108/minute, respiratory rate of 26/minute, and saturation of 90% on room air. Lungs examination was consistent with left sided pleural effusion with normal examination of other systems. Breast examination was normal except mild tenderness on the left chest without any skin dehiscence. Chest radiograph and subsequent computed tomography (CT) of thorax confirmed a moderate to large left pleural effusion and atelectasis of the left lung without any lymph node enlargement or cardiac abnormalities (Figure 1). Full blood examination was normal with no leukocytosis or bandemia. Blood chemistry was normal with mildly elevated AST and ALT. The LDH was 1200 IU/L with ESR of 50 mm/hr and CRP of 17.9 mg/dL. The pro-BNP was 30 pg/mL and Troponin was 0.01 ng/mL with no ST-T changes on EKG.

**Figure 2:**
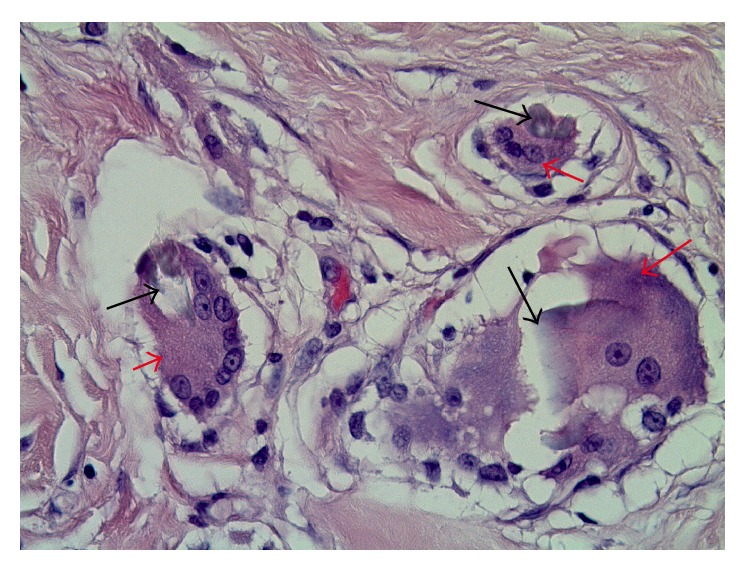
Pathology showing multinucleated giant cells (red arrow) surrounding the silicone particles (black arrows).

The patient was admitted and empirically started on broad spectrum antibiotics. Thoracentesis and placement of pigtail catheter yielded straw colored, cloudy fluid, exudative in nature according to Light's criteria [4]. Fluid examination showed WBC of 7988/mL with lymphocyte predominance, RBC of 18144/mL, LDH of 3855 IU/L, glucose of <20 mg/dL, amylase of 33 U/L, and total protein of 4.9 gm/dL. Fluid Gram staining and cultures were negative for bacteria, fungus, and acid-fast bacilli. Fluid cytology revealed mesothelial cells, macrophages, and lymphocytes with no malignant cells. The chest pain was persistent with reaccumulation of effusion. Repeat cultures were negative with no evidence of sepsis. The concern for foreign body reaction to implants or ruptured or infected implants was raised with subsequent explorative surgery and explantation. Significant inflammation and mild fluid collection were found at the implant site. The pathology was significant for multinucleated giant cells consisting of silicone particles and mononuclear infiltrate suggestive of foreign body reaction, without any malignant cells (Figure 2). Bacterial, fungal, and mycobacterial stains and cultures were negative after six weeks. Patient's symptoms significantly improved after the explantation. She is symptom free after three-month follow-up in the office.

## 3. Discussion

Silicone material has been in use for breast implants since many years. Annually millions of women worldwide undergo silicone gel filled breast implants without any serious complications. Pleural effusion following breast implants is very unusual. The diagnosis of pleural reaction to silicone gel implants is based upon the clinical history, cytopathological examination, and excluding alternative pathology on fluid examination. In our patient with a recent history of breast tumor, malignant pleural effusion should be in the differential as well as infective causes due to recent surgery. Pleural fluid analysis with negative cytology and without any microorganisms and elevated serum inflammatory markers indicated foreign body reaction to silicone gel filled breast implants which was confirmed on pathology. Foreign body reaction (FBR) refers to inflammatory reaction provoked by implanted materials such as medical devices or breast implants [5]. Tissue cells and infiltrated inflammatory cells create a dynamic microenvironment and produce different chemicals such as cytokines, chemokines, and matrix metalloproteinases (MMPs) which in turn mediate FBR [6]. In soft tissues, FBR presents as cellular inflammation and fibrous encapsulation with macrophages [7]. Flessner et al. [8] have demonstrated mesothelial cells, macrophages, fibroblasts, and T cells on the sterile catheters implanted into the rats abdomen for 20 weeks. Abbondanzo et al. [9] studied FBR using 17 paraffin-embedded breast tissues and reported that silicone gel filled implants induced chronic inflammation with abundance of T cells, reactive B-lymphocytes, and macrophages. Similarly, in the present case, cytology of pleural fluid revealed mesothelial cells, macrophages, and lymphocytes with no malignant cells and microorganisms, suggesting that the effusion was the result of FBR to silicone gel filled breast implants.

Although FBR in the form of “fibrous capsule around an implant” is a well-known phenomenon, it rarely causes pleural effusion. Stevens et al. [3] reported a 31-year-old female who presented with history of left sided pleuritic chest pain and pleural effusion after a traumatic rupture of silicone implants. Similar to the present case, Stevens et al. [3] reported slightly turbid and straw colored pleural fluid. However, they reported more protein content (46 gm/L), less glucose (5.3 mmol/L), and less LDH (372 IU/L) than those of the present case. These differences might be due to the chronicity and severity of FBR to silicone gel filled breast implant or rupture of the implant by the blow.

Similarly, Hirmand et al. [10] reported a patient with history of pain in upper back 20 years after bilateral silicone gel breast augmentation. Left sided pleural effusion was found due to ruptured left sided breast implant. All laboratory tests were normal except the presence of silicone scanning electron microscopy. The patient's symptoms resolved after therapeutic thoracentesis. They suggested that silicone might have reached pleural cavity after the rupture of the implant which resulted in subsequent pleural effusion. It indicates that silicone breast implants have potential to induce FBR and pulmonary problems. In the same way, Taupmann and Adler [11] reported pleural effusion caused by iatrogenic breast implant rupture.

The diagnosis is based upon high index of clinical suspicion after excluding the common causes. The authors reported various modalities of imaging to evaluate and diagnose breast implant integrity and rupture [12], but their use has been limited to the availability and the institutional practices. The recurrent effusion in this patient with elevated serum inflammatory markers and the temporal correlation, exclusion of infections, and visualization of inflammation on breast exploration coupled with dramatic improvement of pain and effusion after removal of implants helps in confirming the diagnosis and treatment.

## 4. Conclusion

In summary, silicone gel filled breast implants have potential to provoke chronic inflammation of pleura and subsequent pulmonary complications such as chest pain, dyspnea, and pleural effusions. Although the present case study is a sound addition to the literature, additional studies at a broad level are needed to report and demonstrate conspicuous features of FBR to silicone gel filled breast implants.
